# Rationale and Design of the Leipzig (LIFE) Heart Study: Phenotyping and Cardiovascular Characteristics of Patients with Coronary Artery Disease

**DOI:** 10.1371/journal.pone.0029070

**Published:** 2011-12-22

**Authors:** Frank Beutner, Daniel Teupser, Stephan Gielen, Lesca Miriam Holdt, Markus Scholz, Enno Boudriot, Gerhard Schuler, Joachim Thiery

**Affiliations:** 1 Institute of Laboratory Medicine, Clinical Chemistry and Molecular Diagnostics, University Hospital Leipzig, Leipzig, Germany; 2 Department of Cardiology, Heart Center, University Leipzig, Leipzig, Germany; 3 Institute of Medical Informatics, Statistic and Epidemiology, University Leipzig, Leipzig, Germany; 4 Leipzig Research Center for Civilization Diseases (LIFE), University Leipzig, Leipzig, Germany; University of Tor Vergata, Italy

## Abstract

**Objective:**

We established the Leipzig (LIFE) Heart Study, a biobank and database of patients with different stages of coronary artery disease (CAD) for studies of clinical, metabolic, cellular and genetic factors of cardiovascular diseases.

**Design:**

The Leipzig (LIFE) Heart Study (NCT00497887) is an ongoing observational angiographic study including subjects with different entities of CAD. Cohort 1, patients undergoing first-time diagnostic coronary angiography due to suspected stable CAD with previously untreated coronary arteries. Cohort 2, patients with acute myocardial infarction (MI) requiring percutaneous revascularization. Cohort 3, patients with known left main coronary artery disease (LMCAD).

**Results:**

We present preliminary results of demographics and phenotyping based on a 4-years analysis of a total of 3,165 subjects. Cohort 1 (n = 2,274) shows the typical distribution of elective coronary angiography cohorts with 43% cases with obstructive CAD and 37% normal angiograms. Cohorts 2 and 3 consist of 590 and 301 subjects, respectively, adding patients with severe forms of CAD. The suitability of the database and biobank to perform association studies was confirmed by replication of the CAD susceptibility locus on chromosome 9p21 (OR per allele: 1.55 (any CAD), 1.54 (MI), 1.74 (LMCAD), p<10^−6^, respectively). A novel finding was that patients with LMCAD had a stronger association with 9p21 than patients with obstructive CAD without LMCAD (OR 1.22, p = 0.042). In contrast, 9p21 did not associate with myocardial infarction in excess of stable CAD.

**Conclusion:**

The Leipzig (LIFE) Heart Study provides a basis to identify molecular targets related to atherogenesis and associated metabolic disorders. The study may contribute to an improvement of individual prediction, prevention, and treatment of CAD.

## Introduction

Coronary artery disease (CAD) and its complications such as myocardial infarction and congestive heart failure are projected to remain the leading cause of mortality in the world [1]. CAD is caused by a complex pattern of interaction of genetic factors and life-style. However, individual CAD susceptibility is not well understood. Combined assessment of traditional risk factors, clinical phenotypes, genetic information as well as proteomic and metabolic data is a promising strategy for the assessment of the individual cardiovascular risk – the premise of ‘personalized medicine’ [2].

A significant source of limitation in many previous genetic studies of CAD has been from imprecise phenotypic characterization of cases and controls [3], [4]. Coronary lesions show a wide range of clinical manifestations, from asymptomatic to stable symptomatic disease with stress-induced angina to the point of acute coronary syndrome, myocardial infarction and even cardiac death. This contributes substantially to the heterogeneity of cohorts that have been assessed to date and many of them are a medley of subjects with stable angina, acute coronary syndrome and with a history of myocardial infarction or coronary revascularization. A detailed and highly standardized phenotypic characterization might therefore contribute to a more valid classification of cases and controls. Moreover, a precise assessment of confounders such as life-style factors and concomitant disease might allow for a more appropriate adjustment for these confounders. The latter will also be important for validation of novel biomarkers using e.g. transcriptomic, proteomic or metabolomic analysis [5], [6].

The Leipzig (LIFE) Heart Study represents a biobank and data base with detailed cardiovascular, metabolic and biochemical characterization of patients with different entities of atherosclerotic coronary disease. These include subjects undergoing first-time coronary angiography due to suspected CAD but previously unknown coronary status (cohort 1), subjects with acute myocardial infarction as first manifestation of CAD (cohort 2) and subjects with left main coronary artery disease (cohort 3). The suitability of the database and biobank to perform association analyses will be supported by assessing the CAD susceptibility locus on chromosome 9p21.

## Methods

A detailed description of methods is provided in the online supplement (Data S1).

### Overview

The Leipzig (LIFE) Heart Study is designed as observational study to evaluate biochemical and molecular biomarkers and their ability to assess the presence and severity of CAD in symptomatic subjects (cross-sectional) and to predict the future course of disease (longitudinal). Follow-up at 5-year intervals will provide prospective information about major cardiac clinical events of the initial study subjects (cardiovascular death, myocardial infarction/re-infarction, coronary revascularization). The study meets the ethical standards of the Declaration of Helsinki. It has been approved by the Ethics Committee of the Medical Faculty of the University Leipzig, Germany (Reg. No 276-2005) and is registered with ClinicalTrials.gov (NCT00497887). Written informed consent has been obtained from all participants involved in the study.

### Study cohorts

Cohort 1: Subjects with suspected CAD. Patients included in cohort 1 are referred to coronary angiography by an outpatient cardiologist. Coronary angiography was indicated by clinical symptoms and non-invasive testing. Patients with any previous coronary revascularization in form of percutaneous coronary intervention (PCI) or coronary artery bypass graft (CABG) are excluded to obtain subjects with coronary first-time events and untreated coronary arteries (Table 1).

**Table 1:** Inclusion and exclusion criteria of the Leipzig (LIFE) Heart Study.

	Inclusion criteria	Exclusion criteria
Overall	Age 18–85 years	Severe systemic diseases (e.g. active cancer, autoimmune disease)
	Ability to consent	
Cohort 1	Patients with suspected CAD referred for diagnostic invasive coronary angiography	Coronary revascularization (PCI/CABG) in history
		Acute coronary syndrome
		Severe valvular disease
Cohort 2	Patients with acute myocardial infarction (Troponin T>0.04 and angiographic detectable culprit lesion)	Coronary revascularization (PCI/CABG) prior to current myocardial infarction
Cohort 3	Patients with angiographic ≥50% luminal reduction of the left main coronary artery	

PCI, percutaneous coronary intervention; CABG, coronary artery bypass graft.

Cohort 2: Subjects with myocardial infarction. Patients included in cohort 2 required primary or rescue PCI caused by acute myocardial infarction. Symptom onset and interval to revascularization is limited to a maximum of 60 and 36 hours before enrollment and blood taking, respectively. The rationale is to evaluate circulating biomarkers of the early postinfarction phase and their ability to predict the clinical outcome and risk for future coronary events (Table 1).

Cohort 3: Subjects with left main coronary artery disease (LMCAD). Patients included in cohort 3 have known CAD and significant LMCAD with luminal reduction ≥50% ostial, mid-shaft or at bifurcation. Subjects may be treated conservative or had required revascularization in history (Table 1).

### Data collection

Data collection in the Leipzig (LIFE) Heart Study reverts to standardized, validated instruments and procedures which have been proved of value in epidemiologic and clinical studies. Medical and family history, life style habits and medication are surveyed in a standardized interview. Subjects with suspected CAD pass an extended standardized study program including anthropometric measurements, blood pressure measurement, electrocardiogram, exercise test, echocardiography (Figure S1, Table S1) and ultrasound of carotid (Figure S2) and peripheral arteries besides coronary angiography. Therefore, atherosclerotic burden is estimated in the three mostly affected arterial beds: peripheral, carotid and coronary arteries. Coronary angiography is performed following the standards of the institution. Gensini score index is calculated and patients are classified in sets of subjects with normal angiogram (no CAD), subjects with wall irregularities but <50% luminal reduction (CAD<50%) and subjects with obstructive CAD defined as at least one stenosis of 50% or more in any coronary vessel (CAD≥50%) (Figure S3). Significant LMCAD was defined as visual luminal reduction ≥50% of the left main trunk (Figure S4).

### Laboratory

Blood of subjects with suspected CAD (cohort 1) is drawn before invasive diagnostic, hence unaffected by revascularization procedures and escalation of pharmacological therapy. Blood of subjects with acute myocardial infarction (cohort 2) is drawn within 6 to 36 hours after the interventional revascularization. Blood of patients with LMCAD (cohort 3) is drawn irrespective of earlier coronary procedures. Multiple aliquots of serum, plasma, whole blood, DNA, RNA and peripheral mononuclear blood cells are stored at −80°C or liquid nitrogen for further analysis. Clinical chemistry and a set of metabolic, cardiac and inflammatory marker are measured on the day of blood sampling using an automated Roche Modular analysis system (Roche Diagnostics, Mannheim, Germany). Single nucleotide polymorphisms (SNP) rs10757274, rs2383206, rs2383207 and rs10757278 are genotyped for replication studies of the CAD-susceptibility locus on chr9p21.

### Statistics

The sample size calculation to estimate the suitability of our study for genetic studies is given in the Table S2. SPSS version 16.0 was used for statistical analyses. Categorical data are expressed as numbers and percentages and were compared using the χ^2^ or Fisher's exact test, as appropriate. Continuous variables are expressed as the arithmetic mean ± standard deviation for normally distributed variables and as geometric mean ± standard deviation for non-normally distributed variables. They were compared using a one-way ANOVA and Kruskal-Wallis test with post-hoc test, respectively. *P*-values<0.05 were considered statistically significant. Genetic association analyses were performed using logistic regression techniques. Associations of cardiologic endpoints with single SNPs and the GGGG haplotype were adjusted for age, sex, diabetes and cigarette smoking packyears. CAD phenotypes (except myocardial infarction) were also adjusted for HDL and LDL. Additive models were calculated throughout. Calculations were performed using the statistical software package R (www.r-project.org).

## Results

### Description of study cohorts

Recruitment of subjects started in December 2006 and is ongoing. The current results are based on 3.165 subjects in 3 cohorts with different entities of CAD (Table 2).

**Table 2:** Numbers of subjects recruited in several cohorts and composite phenotypes.

Cohort/Coronary phenotype	N (%)
All	3165 (100)
Cohort 1 (suspected CAD, total)	2274 (72)
- normal angiogram	812 (26)
- non-obstructive CAD^a^	456 (15)
- obstructive CAD^b^	906 (28)
- no angiography	100 (3)
Cohort 2 (myocardial infarction)	590 (19)
Cohort 3 (LMCA disease)	301 (9)
All CAD (obstructive from Cohorts 1, 2, 3)	1797 (57)
Stable CAD (obstructive from Cohorts 1, 3)^c^	956 (30)
Single-vessel-disease (obstructive from Cohorts 1, 2)	635 (20)
Multi-vessel-disease (obstructive from Cohorts 1, 2, 3)	1162 (37)
All myocardial infarction (cohort 1, 2, 3)^d^	841 (27)
All LMCA disease (from Cohorts 1, 2, 3)	492 (16)

a subjects with angiographic coronary wall irregularities <50% luminal reduction.

b subjects with angiographic stenoses ≥50% luminal reduction in at least one major coronary artery.

c subjects of cohort 1 and 3 with normal troponin level, obstructive CAD and no myocardial infarction in their recollected history.

d subjects of cohort 1 and 2 with elevated troponin and angiographically culprit coronary lesion, subjects of cohort 3 with myocardial infarction in their recollected history.

### Cohort 1: Suspected coronary artery disease

**Figure 1:**
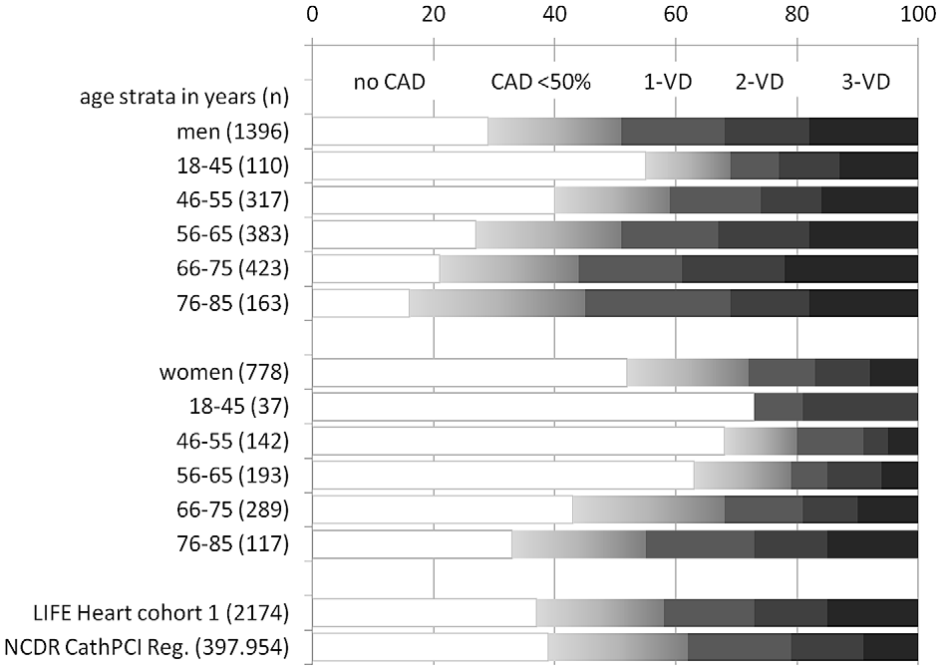
Angiographic findings of subjects with suspected CAD (cohort 1). Arrangement in age strata beginning with 18–45 years followed by 10-years intervals for men and women, separately. Coronary angiograms were interpreted as *no CAD* when angiographically normal coronary arteries were found, the presence of wall irregularities (*WI*) was interpreted as intermediate coronary atherosclerosis (CAD<50%). Stenoses with ≥50% luminal reduction were rated as obstructive CAD and assessed as 1-, 2- or 3-vessel disease (*1VD, 2VD, 3VD*). For comparison, representative results of the American College of Cardiology National Data Registry (NCDR CathPCI Reg.) are shown including patients with elective coronary angiography for suspected coronary artery disease [21].

Cohort 1 consists of 1,457 men and 817 women, who were referred for diagnostic coronary angiography due to suspected coronary artery disease. Completeness of phenotyping was high for blood sampling, laboratory analyses, interview and ECG (100%, respectively), echocardiography (99%), carotid ultrasound (96%) and ABI (95%). An exercise stress test was completed in 56% of the study participants while remaining patients had cardiologic or orthopedic disabilities. Coronary angiography was performed in 97% of study patients (indication for coronary angiography was revised in n = 100 subjects after study enrollment). Angiographically detectable atherosclerosis was present in the coronary tree of 71% of men and 48% of women. The remaining subjects were free of angiographically visible CAD. 49% of males and 29% of females had luminal reduction of ≥50% in at least one major coronary artery, resulting in assignment to percutaneous coronary intervention or coronary artery bypass grafting in 38% of males and 22% of females. 32% of men and 18% of women had a multi-vessel disease. Gensini scores of the male and female subsets with CAD≥50% were 40±32 and 32±28, respectively. Distributions of angiographic findings are shown in Figure 1 as age strata in 10-years intervals. All age strata comprise subjects with various stages of CAD as well as subjects with angiographically normal coronary arteries.

### Cohort 2: Acute myocardial infarction

Cohort 2 consists of 431 men and 159 women with acute myocardial infarction, which were included 6 to 36 hours after PCI. The mean time from PCI to inclusion was 20±8 hours. 70% had an ST-elevation myocardial infarction (CK 36±34 U/l) and remaining patients occurred with non-ST-elevation myocardial infarction (CK 15±14 U/l). Myocardial infarction resulted in a reduction of left ventricular ejection fraction <50% in 48% of STEMI cases and 34% of NSTEMI cases. All subjects with myocardial infarction had an identifiable culprit lesion, in 34% it was localized in the right coronary artery, 48% in the left anterior descending, and 18% in the circumflex or affiliated branches. 52% had a single-vessel-disease while 48% had a multi-vessel disease yet with further significant stenosis besides the culprit lesion. Gensini scores were 40±29 in NSTEMI cases and 45±28 in STEMI cases.

### Cohort 3: Left main coronary artery disease

Cohort 3 consists of 242 men and 59 women with known left main coronary artery disease (≥50% stenosis). Patients were recruited at an average of 6 years after diagnosis (range 0–37 years). Therefore, most of these individuals had already undergone coronary revascularization by PCI (35% ), bypass graft (30%) or both (19%). Average age at first diagnosis of CAD was 63±11 years. Interestingly, 46% of these patients reported a myocardial infarction in their recollected history. According to retrospective angiograms mean value of luminal stenosis was 70±17% (range 50–100%).

### Demographic and cardiovascular risk factors

Demographic and clinical characteristics of study subjects are summarized for each cohort and for men and women, separately (Table 3 and 4). As expected from the earlier onset of CAD, men included in the study were significantly younger than women (62.0±11.6 and 64.7±11.2 years, respectively). Study participants were obese with mean body-mass index >28 kg/m^2^ in all three cohorts. One third of study subjects were categorized as diabetic in both genders. In cohort 1, 21% of men and 11% of women were current smoker and 52% and 16% had a history of smoking, respectively. In cohort 2 the proportion of current smokers was much higher (49% and 36% of men and women, respectively).

**Table 3:** Characteristics of male subjects.

	Cohort 1 - ‘suspected CAD’					Cohort 2 - ‘myocardial infarction’		Cohort 3 - ‘LMCAD’	
					Significance		Significance		Significance
Parameter	Men	No CAD	CAD<50%	CAD≥50%	*No CAD* vs. *CAD≥50%*	Men	vs. *CAD≥50%*	Men	vs CAD*≥50%*
n	1457^a^	405 (28)	311 (22)	680 (47 )		438		242	
Age (years)	61.0±11.2	57.6±11.4	63.3±10.6	63.5±10.6	<0.001	59.4±12.2	<0.001	68.6±9.5	<0.001
BMI	29.6±4.7	29.8±4.9	29.9±5.3	29.4±4.2	ns	28.6±4.2	<0.001	28.3±4.1	0.002
Diabetes	431 (30)	87 (21)	119 (38)	216 (32)	<0.001	88 (20)	<0.001	105 (43)	<0.001
Smoker (former/current)	756/298 (52/21)	220/71 (54/18)	170/63 (54/20)	350/149 (52/22)	ns	127/215 (29/49)	<0.001	154/30 (63/12)	<0.001
Family history	415 (29)	102 (25)	76 (25)	219 (32)	0.047	153 (35)	ns	80 (33)	ns
SBP/DBP (mmHg)	139/84	137/86	141/84	140/83	0.043/0.001				
Gensini score	20.1±29.4	0.0	2.7±2.7	39.9±31.7	<0.001	44.4±29.6			
Total-C	5.60±1.17	5.50±1.10	5.53±1.08	5.67±1.24	0.013	5.11±1.08	0.001	4.44±1.04	<0.001
HDL-C	1.18±0.34	1.23±0.35	1.18±0.37	1.16±0.33	0.008	1.17±0.32	ns	1.12±0.31	0.046
LDL-C	3.62±1.06	3.45±0.96	3.53±0.97	3.75±1.13	<0.001	3.10±0.97	<0.001	2.55±0.83	<0.001
TG	1.81±1.70	1.84±2.43	1.78±1.46	2.10±1.29	ns	1.74±1.09	<0.001	1.82±1.25	0.005
hsTroponin T ng/l	13 (0–2650)	11 (0–1780)	11 (0–480)	14 (0–2650)	<0.001	1574 (10–38280)	<0.001	18 (0–4330)	ns
Nt-proBNP	170±1207	110±1496	157±1132	228±1035	ns	1649±5006	<0.001	497±4083	<0.001
hsCRP mg/l	2.4±11.3	1.9±9.0	2.4±10.4	2.7±12.8	0.013	43±61	<0.001	3.0±9.3	ns
% ASA	52	41	49	60	<0.001	12	<0.001	87	<0.001
% RAAS blocker	69	62	77	70	0.026	31	<0.001	93	<0.001
% Beta blocker	54	51	52	56	ns	19	<0.001	90	<0.001
% Ca antagonist	23	17	28	25	0.011	10	<0.001	28	ns
% Diuretic	37	32	42	37	ns	8	<0.001	59	<0.001
% Statin	35	27	36	39	<0.001	12	<0.001	82	<0.001

BMI, body-mass index in kg/m^2^; SBP/DBP, systolic and diastolic blood pressure in mmHg; Total-C/HDL-C/LDL-C, cholesterol in mmol/l; hsCRP, high sensitive C-reactive protein in mg/l; RAAS, renin-angiotensin-aldosterone-system; ASA, acetylsalicylic acid. Values are given as arithmetic mean ± standard deviation for normally distributed parameters, geometric mean ± standard deviation for non normal distributed parameters or as percent of subjects for categorical items. Statistic differences between no CAD and CAD ≥50 were calculated using Students t-test/Kruskal-Walis test for scaled items and Chi-square test for categorical items. ^a^ including 61 men without angiography.

**Table 4:** Characteristics of female subjects.

	Cohort 1 - ‘suspected CAD’					Cohort 2 - ‘myocardial infarction’		Cohort 3 - ‘LMCAD’	
					Significance		Significance		Significance
Parameter	Women	No CAD	CAD<50%	CAD≥50%	*No CAD* vs. *CAD≥50%*	Women	vs. *CAD≥50%*	Women	vs CAD*≥50%*
n	817^a^	407 (50)	145 (18)	226 (28)		152		59	
Age (years)	64.3±10.8	61.6±10.7	67.4±8.8	66.7±11.0	<0.001	64.8±13.2	ns	70.4±10.4	ns
BMI	30.2±5.5	29.8±5.4	30.9±5.5	30.3±5.6	ns	28.7±4.9	0.010	28.0±4.7	0.008
Diabetes	239 (29)	89 (22)	54 (37)	88 (39)	<0.001	46 (30)	0.071	32 (54)	0.026
Smoker (former/current)	134/93 (16/11)	63/36 (15/9)	22/18 (15/12)	43/36 (19/16)	0.043	20/55 (13/36)	<0.001	14/3 (24/5)	ns
Family history	296 (36)	133 (33)	69 (48)	80 (36)	ns	47 (29)	ns	17 (30)	ns
SBP/DBP (mmHg)	136/83	135/85	140/84	137/80	ns/<0.001				
Gensini score	9.8±20.8	0	2.4±2.6	32.3±27.9	<0.001	41.4±24.3	<0.001		
Total-C	5.87±1.16	5.73±1.04	5.89±1.16	6.12±1.31	0.013	5.00±1.13	0.001	4.81±1.19	<0.001
HDL-C	1.48±0.40	1.54±0.40	1.42±0.37	1.43±0.39	0.006	1.18±0.34	<0.001	1.34±0.44	ns
LDL-C	3.71±1.10	3.53±0.97	3.75±1.17	3.99±1.23	<0.001	2.98±0.91	<0.001	2.69±0.99	<0.001
TG	1.76±0.90	1.66±0.87	1.93±1.11	1.81±0.81	ns	1.71±1.11	ns	1.77±0.98	ns
Troponin T ng/l	11 (0–1420)	10 (0–50)	11 (0–180)	14 (0–1420)	<0.001	1709 (50–31580)	<0.001	18 (0–4790)	ns
Nt-proBNP	188±1405	147±659	184±638	317±2410	0.001	1732±4068	<0.001	499±1441	ns
hsCRP mg/l	2.7±7.2	2.3±5.6	2.9±8.0	3.5±8.9	<0.001	44±63	<0.001	3.7±24.0	ns
% ASA	53	45	53	68	<0.001	13	<0.001	80	<0.001
% RAAS blocker	68	65	70	74	0.039	63	0.026	97	<0.001
% Beta blocker	65	62	73	65	ns	38	<0.001	97	<0.001
% Ca antagonist	26	19	37	30	0.010	33	ns	38	0.026
% Diuretic	43	37	52	48	0.019	42	ns	62	<0.001
% Statin	36	27	45	46	<0.001	25	<0.001	88	<0.001

See legend to Table 3. ^a^ including 39 women without angiography.

Comparison of subjects with angiographically normal and diseased coronary arteries showed an expected distribution of traditional risk factors for both genders. CAD-patients were older, smoked more, were more often diabetic, had lower levels of HDL, higher levels of LDL and higher levels of C-reactive protein (Table 3 and 4). Interestingly, the majority of subjects in cohort 1 were already treated with acetylsalicylic acid (52%), renin angiotensin aldosteron system inhibitors (69%), betablocker (58%), Calcium antagonists (24%), diuretics (39%) and statins (35%) before invasive coronary diagnostics.

### Replication study of CAD susceptibility locus on Chromosome 9p21

**Figure 2:**
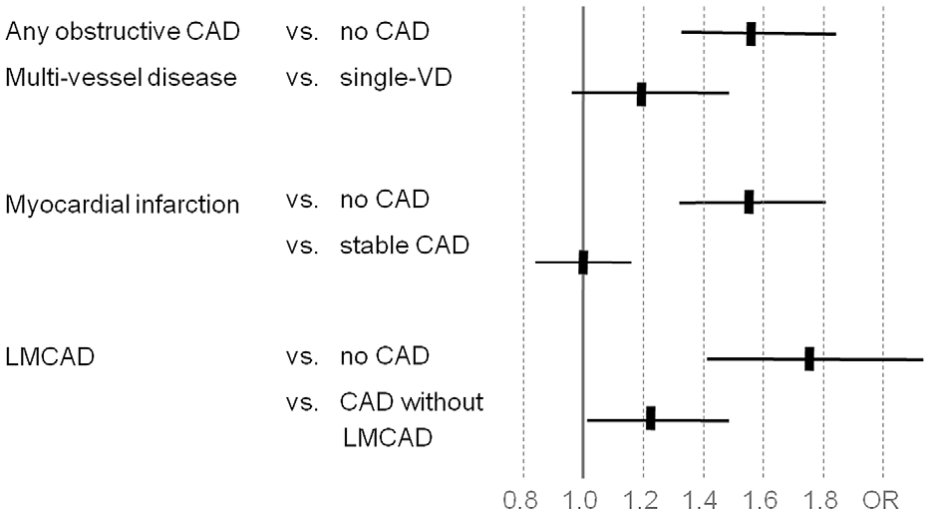
Case-control based association and within-CAD association at chromosome 9p21^a^. ^a^based on haplotype block consisting of 4 SNPs, myocardial infarction defined as elevated troponin and culprit coronary lesion at time of recruitment (cohort 1 and 2) or recollected myocardial infarction (cohort 3), stable CAD defined as obstructive CAD, normal troponin level at time of recruitment and no recollected myocardial infarction (cohort 1 and 3).

To assess the suitability for performing genetic association studies, we finally tested for an association of CAD severity with the chromosome 9p21 genotype. The latter had been identified as the most robust locus associated with coronary artery disease and myocardial infarction in recent genome-wide association studies [7]–[10]. Replication of the chromosome 9p21 locus has been suggested as quality control for further genetic studies of cardiovascular disease [11]. We genotyped SNPs rs10757274, rs2383206, rs2383207 and rs10757278 of the CAD-risk haplotypic block on chromosome 9p21 (call rates 99%, 97%, 99% and 98%, respectively). The risk allele (G) frequencies were 50%, 52%, 53% and 49%, respectively, and were in strong linkage disequilibrium (minimum of pairwise R^2^ = 0.83, Figure S5). G-alleles of all four SNPs were associated with obstructive CAD, myocardial infarction and LMCAD when comparing diseased subjects to those with normal angiograms (Figure 2). The odds ratios of the risk-haplotype were between 1.54 to 1.74 per allele (p-values 7.1×10^−8^ to 6.9×10^−7^) for all these forms of CAD using a logistic regression analysis under the assumption of an additive model and after adjustment for the traditional risk factors (Table 5). To assess the effect of the 9p21 locus between different forms of CAD, we next performed associations within CAD cohorts. Comparing subjects with stable obstructive CAD to subjects with myocardial infarction, no association with 9p21 variants was seen (OR 1.00, CI 0.87–1.15). In contrast, patients with severe forms of CAD such as LMCAD had a stronger association with 9p21 than patients with obstructive CAD without LMCAD (OR 1.22, CI 1.01–1.48), even though it must be noted that these results were at the margin of statistical significance (p = 0.042; Figure 2, Table 6).

**Table 5:** Case-control based association of chromosome 9p21 with CAD.

Objective	Myocardial infarction			LMCAD			All CAD		
Control	no CAD (812)			no CAD (812)			no CAD (812)		
Case	Myocardial infarction (841)			LMCAD (492)			Obstructive CAD (1797)		
Variant	OR	(95% CI)	p	OR	(95% CI)	p	OR	(95% CI)	p
rs10757274	1.53	(1.32–1.77)	1.2×10^−8^	1.74	(1.42–2.14)	1.4×10^−7^	1.56	(1.33–1.83)	3.0×10^−8^
rs2383206	1.53	(1.32–1.78)	2.2×10^−8^	1.76	(1.43–2.17)	1.2×10^−7^	1.58	(1.35–1.85)	1.2×10^−8^
rs2383207	1.51	(1.30–1.75)	5.5×10^−8^	1.69	(1.38–2.08)	5.7×10^−7^	1.53	(1.31–1.79)	1.3×10^−7^
rs10757278	1.46	(1.26–1.69)	5.2×10^−7^	1.65	(1.34–2.04)	2.1×10^−6^	1.47	(1.25–1.72)	2.2×10^−6^
GGGG haploblock	1.54	(1.32–1.80)	7.1×10^−8^	1.74	(1.40–2.16)	6.9×10^−7^	1.55	(1.31–1.84)	2.8×10^−7^

Ordinary logistic regression in an additive model was calculated for combined subsets of CAD adjusted to major risk factors of atherosclerosis (age, gender, smoking, diabetes, HDL-C- and LDL-C-levels adjusted for statin treatment). Analyses of myocardial infarction were only adjusted for age, gender, smoking, diabetes, but not for HDL-C and LDL-C, because the latter parameters significantly change during the acute event. ORs are given per allele.

**Table 6:** Within CAD association of chromosome 9p21 with subcategories of CAD.

Objective	Myocardial infarction			LMCAD			Multi-vessel disease		
Control	Stable CAD≥50% (956)			CAD≥50% without LMCAD (1305)			Single-vessel disease (635)		
Case	Myocardial infarction (841)			LMCAD (492)			Multi-vessel disease (1162)		
Variant	OR	(95% CI)	p	OR	(95% CI)	p	OR	(95% CI)	p
rs10757274	1.01	(0.88–1.15)	0.94	1.24	(1.03–1.49)	0.020	1.12	(0.92–1.34)	0.278
rs2383206	0.99	(0.86–1.13)	0.87	1.21	(1.01–1.46)	0.039	1.12	(0.98–1.46)	0.074
rs2383207	1.01	(0.88–1.16)	0.89	1.20	(1.01–1.44)	0.044	1.20	(0.98–1.46)	0.074
rs10757278	1.01	(0.88–1.16)	0.87	1.24	(1.03–1.48)	0.022	1.20	(0.99–1.47)	0.069
GGGG haploblock	1.00	(0.87–1.15)	0.98	1.22	(1.01–1.48)	0.042	1.19	(0.97–1.47)	0.097

See legend to Table 5.

## Discussion

Here, we present the study design and data of 3.165 subjects enrolled in the ongoing Leipzig (LIFE) Heart Study. The study includes subjects with different severity and courses of coronary artery disease. CAD was prevalent in 78% of males and 57% of females, while the remaining subjects were free of angiographically detectable CAD. Patients with obstructive CAD had the expected higher level of conventional risk factors and confirmation of the chromosome 9p21 risk locus indicates excellent power for genetic association studies. Moreover, different entities of CAD included in the study allowed detailed analyses within CAD populations.

Cohort 1 of the Leipzig (LIFE) Heart Study presents a typical cohort of patients undergoing elective coronary angiography for suspected CAD. In this cohort, 43% of individuals had obstructive CAD while 37% were free of angiographically visible CAD. These data are comparable with the NCDR CathPCI Registry of 397.954 individuals [12], where 38% had obstructive CAD and 39% no CAD (Figure 1). The selection of cases and controls in genetic studies of CAD is a matter of ongoing discussion. Recent genome-wide association studies of CAD and MI included patients based on retrospective data from CAD/MI-registries, retrospective selection of premature familial CAD/MI cases and subjects from several cross sectional studies with angiographically determined CAD [7]–[10], [13]. The controls used in some of these studies were recruited from study cohorts with non-cardiovascular disease such as neurological, psychiatric, infectious disease and cancer or subjects from population-based studies or healthy blood donors. Only few studies used angiographically determined controls without CAD for some of their replications [7], [8].

The ‘ideal’ control would be a >90 years old individual with no prior history of CAD and completely normal coronary arteries.^4^ However, this design appears unrealistic and would not permit the collection of sufficiently large cohorts needed for detection of small effects or even gene-interactions. Therefore, Luo et al. presented apparently less stringent criteria for phenotype categorization [3]: Controls should be characterized by normal coronary arteries by conventional coronary angiography or multidetector computer tomography (CT), no family history of CAD, no history of cerebrovascular or peripheral artery disease and age of controls much greater than cases (10–20 years). In contrast, cases for coronary artery disease and myocardial infarction, respectively, should present with at least one angiographic stenosis ≥70% in a major epicardial artery, family history of CAD, no history of smoking, no diabetes, normal or low LDL-cholesterol, high HDL-cholesterol and normal C reactive protein. However, today no study exists that meets these criteria. From the total of 2274 subjects included in the untreated and unaffected cohort 1, only 1.5% and 8.5% fulfilled the complete set of criteria suggested by Luo et al for CAD cases and controls, respectively. A recent study has investigated the impact of heterogeneity of phenotype definition of CAD on genetic association studies [14]. Remarkably, these investigators found that differences in phenotype definition make only a small contribution to between study heterogeneity. As a limitation, these results were mostly based on studies of candidate genes, which did not play a role in recent genome-wide association studies of CAD. The authors conclude that even though utility of a clear phenotype definition of CAD might be limited for primary analyses, it might be helpful for secondary analyses after an association with CAD has been established [14].

The mode of recruitment of including only subjects referred to coronary angiography for suspected CAD in cohort 1 is unique to the Leipzig (LIFE) Heart Study and has the advantage of simultaneous collection of cases and controls under equal conditions. Other large angiographic studies have broader inclusion criteria and also recruit patients with non-atherosclerotic indications for coronary angiography such as valvular disease [15]. Only few studies perform combined examinations of the three mostly affected regions of atherosclerosis development such as coronary, carotid and peripheral arteries. These studies use computed tomography of coronary calcium as a surrogate parameter for coronary atherosclerosis [16]–[18]. To our knowledge, the Leipzig (LIFE) Heart Study is currently the only large-scale cohort providing angiographic assessment of coronary arteries combined with sonographic evaluation of carotid and peripheral atherosclerosis. Another important advantage is the highly standardized preanalytical process of blood sampling prior to interventional or surgical treatment of coronary stenosis. This enables valid analytical studies of blood cell gene-expression [19] as well as profiling of mediators and biomarkers of metabolism, atherosclerotic wall pathology and myocardial function, yet unaffected by revascularization procedures and initiation of intensive pharmacological treatment.

Limitations of selecting ‘controls’ from patients presenting with angiographically normal coronary arteries might be the presence of atherosclerotic lesions without affecting arterial lumen and patients that will develop significant lesions and coronary events in future. Angiographic luminography is not suitable to detect outward remodeling of plaques or fatty streak lesions and small fibroatheroma which often represent vulnerable plaques. In spite of these limitations, the classification of cases and controls in the Leipzig (LIFE) Heart Study according to angiographic findings led to an initial cohort with sufficient power to confirm the chromosome 9p21 locus for CAD and verifies the ability to perform genetic association studies. The odds ratios between 1.47 (CI 1.25–1.72) to 1.58 (CI 1.35–1.85) per risk allele of 4 SNPs on chromosome 9p21 are within the range of previous work such as the WTCCC CAD cohort (OR 1.47, CI 1.27–1.70) [20] and a recent meta-analysis (OR 1.21 and 1.35, depending on age) [21], confirming the high discriminatory power of our approach. Assuming that the finally available number of subjects will be >6,000, that number would be adequate for genetic analysis to detect low to intermediate effects (OR 1.2) for common SNPs (MAF>20%). For analysis of very low effects (OR<1.2) or less frequent variants (MAF<10%) pooling with other studies providing similar phenotypes will be necessary (Table S2). The Leipzig (LIFE) Heart Study has already been used for such a strategy [22].

Another advantage is the availability of cohorts with different entities of CAD. This also allowed analyses within CAD populations. Interestingly, we found that patients with LMCAD had a stronger association with chromosome 9p21 than patients with obstructive CAD without LMCAD.

This finding is supported by studies displaying a higher heritability of left main and proximal CAD [23], [24]. In contrast, comparing subjects with stable obstructive CAD to subjects with myocardial infarction, no association with 9p21 variants was seen. The latter finding is supported by previous work, showing that 9p21 did not associate with myocardial infarction once stratified for disease severity [25]. Even though these results show the general suitability of the study for analyses of CAD subtypes, larger sample sizes will be needed to obtain more robust results.

In conclusion, the design and recruitment mode of the Leipzig (LIFE) Heart Study provides a promising basis for innovative molecular and diagnostic studies in patients with coronary artery disease. The identification of new potential targets for lifestyle and pharmacological interventions may lead to an improvement of prevention, diagnosis and treatment of atherosclerotic diseases.

## Supporting Information

Figure S1 **Standardized echocardiographic examination.** PLAX – parasternal long axis; PSAX – parasternal short axis; ALAX – apical long axis; 2,- 3CV – 2-, 3-chamber view, RVOT – right ventricular outflow tract; LVOT – left ventricular outflow tract, TVI – tissue velocity imaging, spectral TDI – spectral tissue doppler imaging; AV – aortic valve; MV – mitral valve; PV – pulmonary valve; TV – tricuspidal valve.
(TIF)

Figure S2 **Standardized sonographic examination of carotid arteries.** CCA – common carotid artery, ICA – internal carotid artery, ECA – external carotid artery, BFI – B-flow imaging.
(TIF)

Figure S3 **Categorization in** ***no CAD*** **,** ***CAD<50%*** **and** ***CAD≥50%*** **according to visuell estimation of lumen narrowing illustrated by proximal and medial segments of the right coronary artery in left-anterior (LAO) and right-anterior (RAO) projections.**
(TIF)

Figure S4 **Schematic of the left main trunk (A).** Luminal reduction ≥50% in the area shaded in red was defined as LMCAD. Representative images showing angiographically normal (B) and a obstructed (C) left main trunks.
(TIF)

Figure S5 **Chromosome 9p21 tagging SNPs (rs10757274, rs2383206, rs2383297, rs10757278) – region (left) and correlation (R^2^) in the Leipzig (LIFE) Heart Study (right). Modified from Holdt et al.** [**7**] **.**
(TIF)

Table S1 **Echocardiographic measurements and calculations.**
(DOCX)

Table S2 **Sample size calculation for genetic association studies.**
(DOCX)

Data S1 **Supporting information with a detailed description of methods.**
(DOCX)
