# Supplementary figures and images for: Rationale and Design of the Leipzig (LIFE) Heart Study: Phenotyping and Cardiovascular Characteristics of Patients with Coronary Artery Disease
Source: PLoS One. 2011 Dec 22;6(12):e29070. doi: 10.1371/journal.pone.0029070 (PMC3245257; doi:10.1371/journal.pone.0029070)

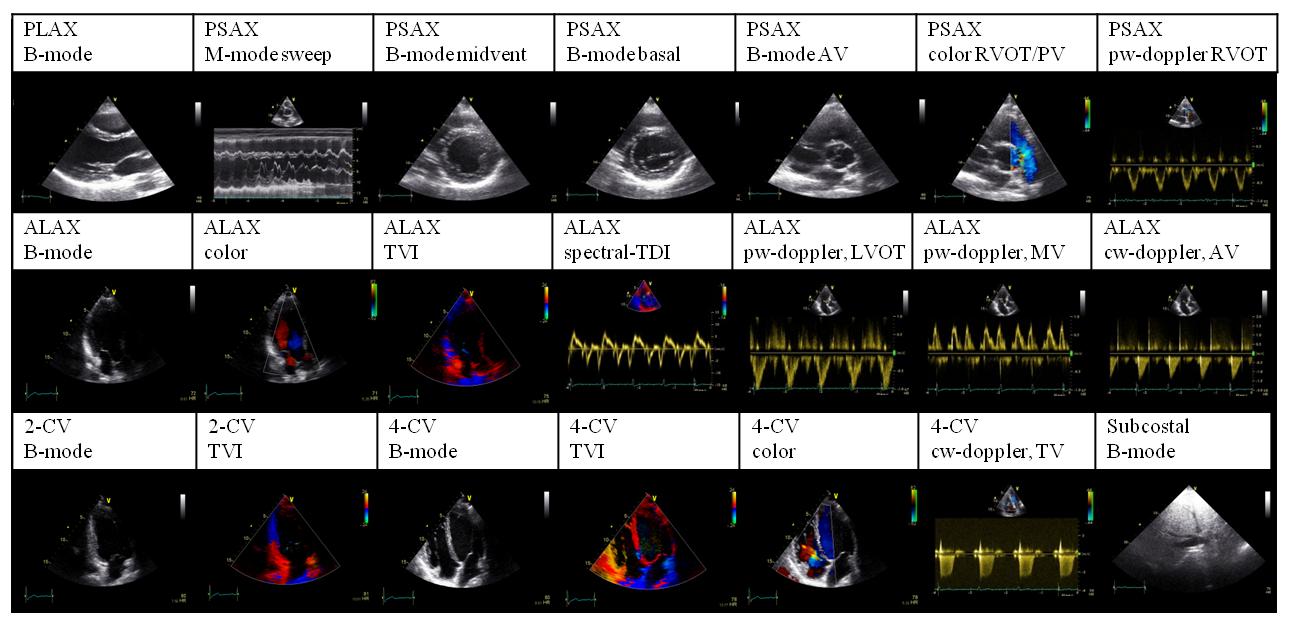

Supplement: Figure S1 — Standardized echocardiographic examination. PLAX – parasternal long axis; PSAX – parasternal short axis; ALAX – apical long axis; 2,- 3CV – 2-, 3-chamber view, RVOT – right ventricular outflow tract; LVOT – left ventricular outflow tract, TVI – tissue velocity imaging, spectral TDI – spectral tissue doppler imaging; AV – aortic valve; MV – mitral valve; PV – pulmonary valve; TV – tricuspidal valve. (TIF) [file pone.0029070.s001.tif]

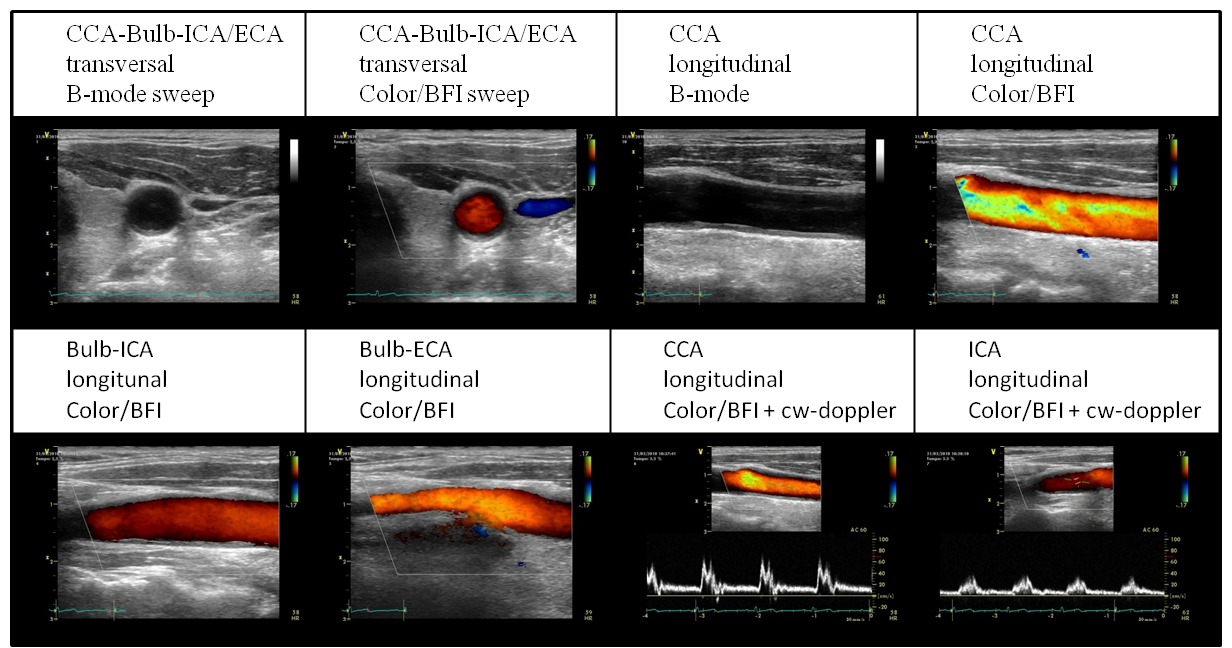

Supplement: Figure S2 — Standardized sonographic examination of carotid arteries. CCA – common carotid artery, ICA – internal carotid artery, ECA – external carotid artery, BFI – B-flow imaging. (TIF) [file pone.0029070.s002.tif]

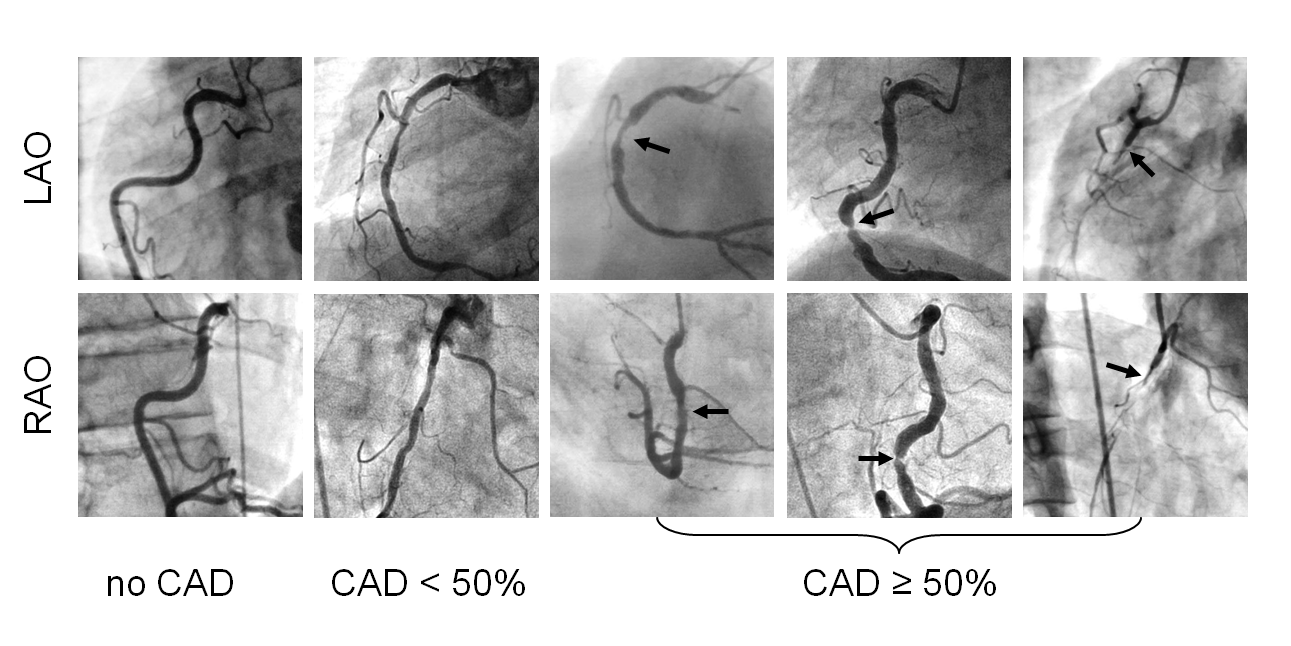

Supplement: Figure S3 — Categorization in no CAD , CAD<50% and CAD≥50% according to visuell estimation of lumen narrowing illustrated by proximal and medial segments of the right coronary artery in left-anterior (LAO) and right-anterior (RAO) projections. (TIF) [file pone.0029070.s003.tif]

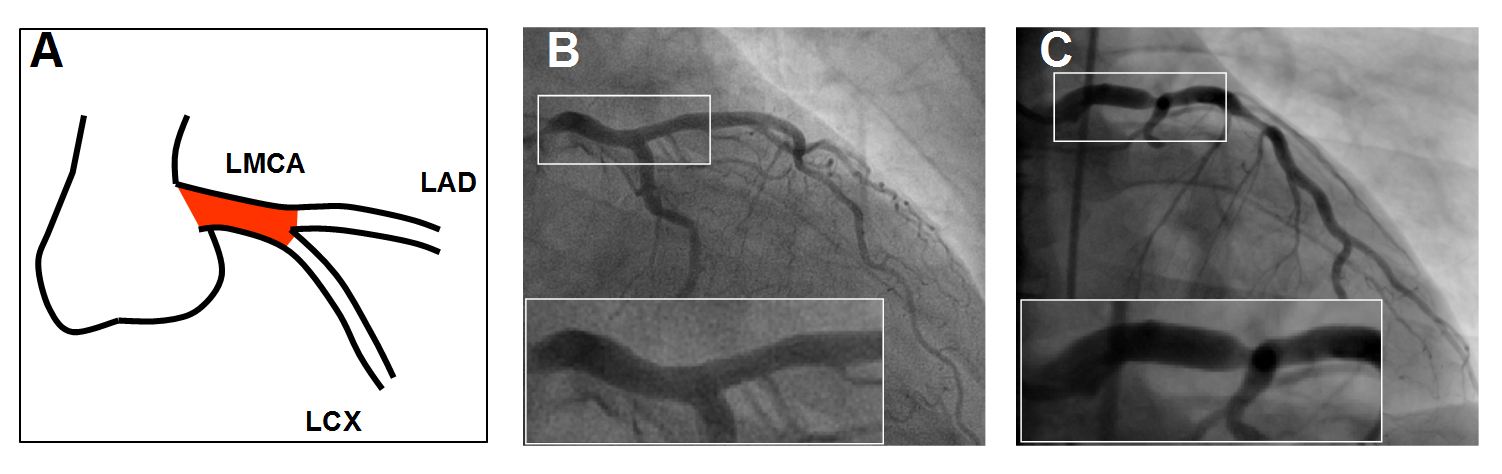

Supplement: Figure S4 — Schematic of the left main trunk (A). Luminal reduction ≥50% in the area shaded in red was defined as LMCAD. Representative images showing angiographically normal (B) and a obstructed (C) left main trunks. (TIF) [file pone.0029070.s004.tif]

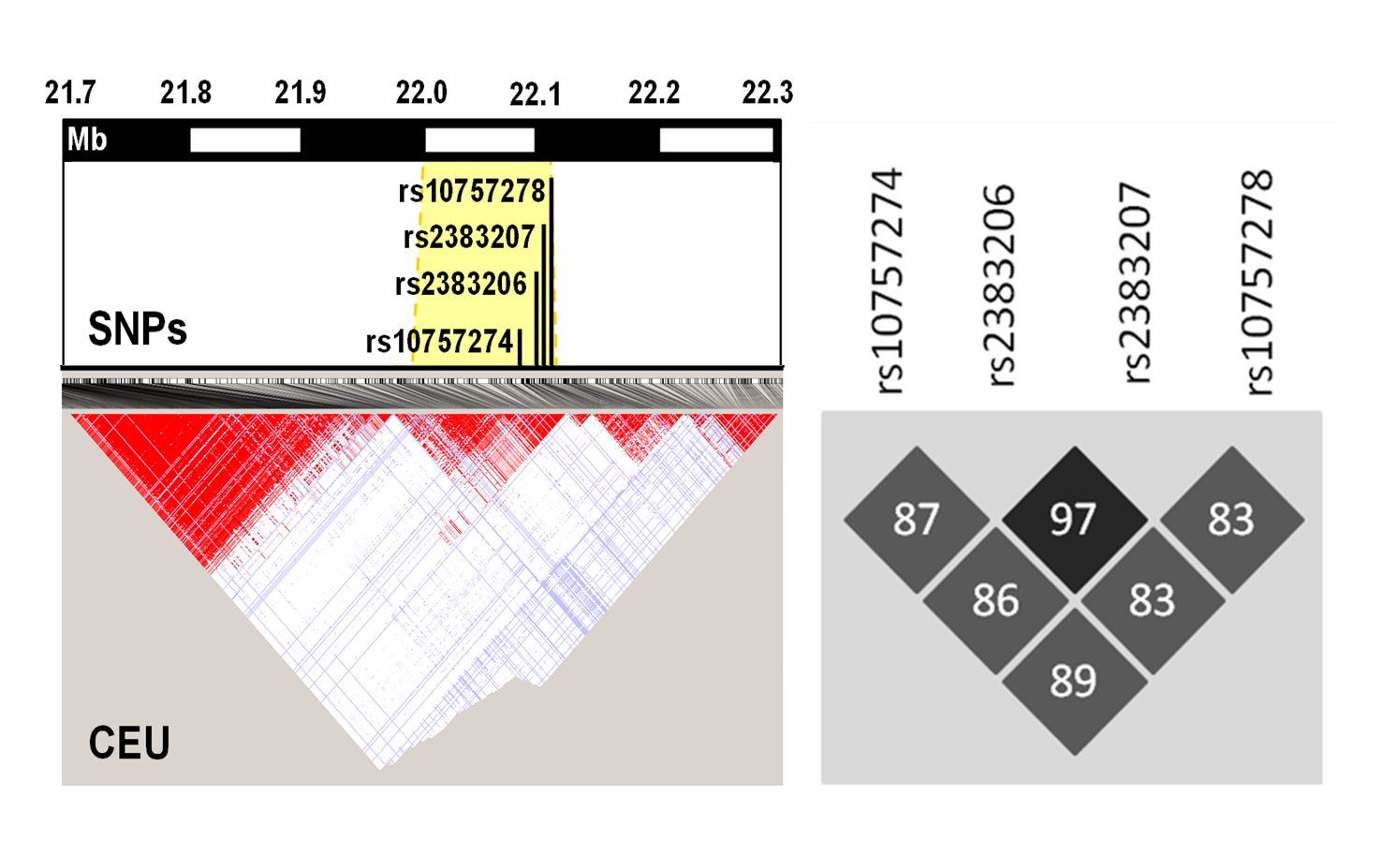

Supplement: Figure S5 — Chromosome 9p21 tagging SNPs (rs10757274, rs2383206, rs2383297, rs10757278) – region (left) and correlation (R2) in the Leipzig (LIFE) Heart Study (right). Modified from Holdt et al. [7] . (TIF) [file pone.0029070.s005.tif]
