# Supplementary material for: Rationale and Design of the Leipzig (LIFE) Heart Study: Phenotyping and Cardiovascular Characteristics of Patients with Coronary Artery Disease
Source: PLoS One. 2011 Dec 22;6(12):e29070. doi: 10.1371/journal.pone.0029070 (PMC3245257; doi:10.1371/journal.pone.0029070)
Supplement: Table S1 — Echocardiographic measurements and calculations. (DOCX) [file pone.0029070.s006.docx]

**Supplemental Table S1.** Echocardiographic measurements and calculations.

| **View** | **Measures** | **Calculation** |
| --- | --- | --- |
| Parasternal short axis (PSAX) Mode-Sweep | Left ventricular diameter endsystolic/enddiastolic | Left ventricular volume endsystolic/enddiastolic |
|  | Thickness of septal/posterior wall endsystolic/ endiastolic | Ejection fraction (Teichholz) |
|  | Aortic diameter | Left ventricular mass |
|  | Left atrium diameter |  |
|  | LVOT diameter |  |
| Apical long axis (ALAX) | Transmitral flow | Aortic valve opening |
|  | Aortic and LVOT flow | Cardiac output |
|  | Tissue doppler anteroseptal |  |
| Apical 2-chamber view | Left ventricular area | Left ventricular volume |
|  | Left atrial area | Left atrial volume |
| Apical 4-chamber view | Left ventricular area | Left ventricular volume |
|  | Left atrial area | Left ventricular ejection fraction (Simpson) |
|  | Tissue doppler septal/lateral | Left atrial volume |
