# Supplementary material for: Rationale and Design of the Leipzig (LIFE) Heart Study: Phenotyping and Cardiovascular Characteristics of Patients with Coronary Artery Disease
Source: PLoS One. 2011 Dec 22;6(12):e29070. doi: 10.1371/journal.pone.0029070 (PMC3245257; doi:10.1371/journal.pone.0029070)
Supplement: Table S2 — Sample size calculation for genetic association studies. (DOCX) [file pone.0029070.s007.docx]

**Supplemental Table S2.** Sample size calculation for genetic association studies.

|  |  | Case-control comparison | | |  | Analysis of quantitative parameters (mean differences) | | |
| --- | --- | --- | --- | --- | --- | --- | --- | --- |
| Minor allele frequency in control group | p-level | OR=1.2 | OR=1.3 | OR=1.5 |  | 0.1*SD | 0.2*SD | 0.3*SD |
| 10% | 0.05 | 2,974 | 1,418 | 582 |  | 5,102 | 1,276 | 570 |
|  | 10^-2^ | 4,393 | 2,089 | 852 |  | 7,588 | 1,897 | 845 |
|  | 10^-3^ | 6,391 | 3,033 | 1,233 |  | 11,096 | 2,775 | 1,234 |
|  | 10^-4^ | 8,362 | 3,963 | 1,609 |  | 14,550 | 3,638 | 1,617 |
| 20% | 0.05 | 2,050 | 992 | 418 |  | 3,407 | 852 | 382 |
|  | 10^-2^ | 3,028 | 1,460 | 612 |  | 5,072 | 1,269 | 569 |
|  | 10^-3^ | 4,406 | 2,121 | 885 |  | 7,413 | 1,855 | 825 |
|  | 10^-4^ | 5,764 | 2,771 | 1,155 |  | 9,722 | 2,433 | 1,083 |
| 30% | 0.05 | 1,943 | 951 | 409 |  | 3,143 | 787 | 353 |
|  | 10^-2^ | 2,870 | 1,401 | 600 |  | 4,674 | 1,169 | 525 |
|  | 10^-3^ | 4,175 | 2,034 | 868 |  | 6,836 | 1,709 | 762 |
|  | 10^-4^ | 5,463 | 2,658 | 1,132 |  | 8,965 | 2,242 | 997 |
