# Supplementary material for: Rationale and Design of the Leipzig (LIFE) Heart Study: Phenotyping and Cardiovascular Characteristics of Patients with Coronary Artery Disease
Source: PLoS One. 2011 Dec 22;6(12):e29070. doi: 10.1371/journal.pone.0029070 (PMC3245257; doi:10.1371/journal.pone.0029070)
Supplement: Data S1 — Supporting information with a detailed description of methods. (DOCX) [file pone.0029070.s008.docx]

**Supporting Information (Data S1)**

**Rationale and design of the Leipzig (LIFE) Heart Study: Phenotyping and cardiovascular characteristics of patients with coronary artery disease**

**Beutner/Teupser et al.**

***Overview***

The study is designed as mono-centric observational study recruiting patients with different entities of CAD: 1) patients undergoing first-time diagnostic coronary angiography for suspected stable CAD, 2) patients with acute myocardial infarction and 3) patients with known CAD and left main coronary artery disease (LMCAD). Blood samples are collected for biochemical and molecular analyses to study the variance of severity and course of CAD development as well as the transition to acute corornary syndromes. Planned long-term follow-up in 5-year intervals will provide prospective information about major cardiac clinical events of the initial study subjects (cardiovascular death, myocardial infarction/re-infraction, coronary revascularization). The Leipzig (LIFE) Heart Study is conducted by the Institute of Laboratory Medicine, Clinical Chemistry and Molecular Diagnostics (ILM), University Hospital Leipzig and the Heart Center of the University Leipzig. The study meets the ethical standards of the Declaration of Helsinki. It has been approved by the Ethics Committee of the Medical Faculty of the University Leipzig, Germany (Reg. No 276-2005) and is registered with ClinicalTrials.gov (NCT00497887). All participants gave written informed consent, also with regard to genetic analyses and long-term storage of biosamples.

***Study cohort and recruitment procedure***

Recruitment of the Leipzig (LIFE) Heart Study started in 2006 and is ongoing. Participants are selected from the daily clinical routine of the Heart Center University Leipzig, Germany. Caucasian men and women are included.

Cohort 1: Subjects with suspected CAD. Patients included in cohort 1 are referred to coronary angiography by an outpatient cardiologist. Coronary angiography was indicated by clinical symptoms and non-invasive testing. Patients with any previous coronary revascularization in form of percutaneous coronary intervention (PCI) or coronary artery bypass graft (CABG) are excluded to obtain subjects with coronary first-time events and untreated coronary arteries. The rationale of this mode of recruitment is also to obtain a larger fraction of subjects with non-diseased coronary arteries as control in addition to the fractions of subjects with different stages of CAD.

Cohort 2: Subjects with myocardial infarction. Patients included in cohort 2 required primary or rescue PCI caused by acute myocardial infarction. Symptom onset and interval to revascularization is limited to a maximum of 60 and 36 hours before enrollment and blood taking, respectively. The rationale is to evaluate circulating biomarkers of the early postinfarction phase and their ability to predict the clinical outcome and risk for future coronary events.

Cohort 3: Subjects with left main coronary artery disease (LMCAD). Patients included in cohort 3 have known CAD and significant LMCAD with luminal reduction ≥ 50 % ostial, mid-shaft or at bifurcation. Subjects may be treated conservative or had required revascularization in history.

***Data collection***

Data collection in the Leipzig (LIFE) Heart Study reverts to standardized, validated instruments and procedures which have been proved of value in epidemiologic and clinical studies. Elaborate study examinations of cohort 1 include electrocardiogram at rest, exercise test, echocardiogram and ultrasound of cerebrovascular and peripheral arteries, which are performed according to standard operating protocols, respectively, before coronary angiography. Interview items, anthropometric and cardiovascular examination procedures were selected and adapted from the ARIC Study [1], the Coronary Artery Surgery Study [2], the Havard Alumni Heart Study [3], the MONICA/KORA Study [4], CARLA Study [5] and the EPIC Study [6]. Subjects included in cohort 2 and 3 complete a standardized interview comparable with cohort 1, further cardiovascular measurements are based on examinations of the clinical setting. Blood is drawn after inclusion criteria are verified and informed consent is obtained. Blood draw occurs before angiography in cohort 1, temporally delayed after PCI in cohort 2 and irrespective of earlier coronary procedures in cohort 3.

***Laboratory***

Standardized blood collecting systems (Sarstedt, Germany; Becton Dickinson, USA) are used for taking various peripheral venous blood samples from each participant: blood for serum, EDTA-anticoagulated blood for hematology, plasma and DNA extraction, citrate-anticoagulated blood for plasma and cell isolation, stabilized whole blood for RNA extraction. Isolation of peripheral blood mononuclear cells (PBMC) for RNA extraction and lymphoblastoid cell-line immortalization is performed using Cell Preparation Tubes (CPT, Becton Dickinson, USA). Centrifugation of serum and PBMC samples is arranged between 30-60 minutes after blood collection. Samples are then stored at 4°C and transported within 5 hours to the Institute of Laboratory Medicine of the University of Leipzig. The laboratory has been accredited according to the accreditation norms ISO 15180 and ISO 17025. Clinical Chemistry, lipids and a set of metabolic, cardiac and inflammatory marker are measured immediately using a Roche Modular analyzer (Roche Diagnostics, Germany). The following parameters are determined: Alanine aminotransferase, alkaline phosphatase, apolipoporotein A, apolipoprotein B, aspartate aminotransferase, bilirubin, BUN, cholesterol, creatine kinase, creatine kinase MB, creatinine, C-reactive protein (high sensitive assay), gamma glutamyltransferase, glucose, haemoglobin A1c, HDL-cholesterol, LDL-cholesterol, small-dense LDL-cholesterol, Lp(a), NT-pro-brain natriuretic peptide, proteine, triglycerides, thyroid-stimulating hormone, troponin T. Hematology parameters and a differential blood count is determined using a Sysmex X-CLASS analyzer (Sysmex, Germany). DNA is extracted from whole blood samples by Invisorb Spin Blood Maxi Kit (Invitek, Germany), dissolved in distilled water and stored at −20°C until analysis. Whole blood RNA is collected stabilized and isolated using the PAXgene Blood RNA Tube and PAXgene 96 Blood RNA Kit (QIAGEN/Becton Dickinson, USA). Multiple aliquots of serum, plasma, whole blood, RNA and PBMCs are stored at -80°C or liquid nitrogen for further analysis.

Single nucleotide polymorphisms (SNP) rs10757274, rs2383206, rs2383207 and rs10757278 are genotyped in all subjects using a homogenous fluorescent method as previously described [7].

***Interview***

The computer-assisted interview is programmed with the Access-based DIAMON interview software which was also used in the MONICA/KORA Study and CARLA Study [8]. Information on medical history, life style, family history and medication are collected in a thirty minutes face-to-face interview by trained study nurses. Selected items of the MONICA/KORA Study interview are used to evaluate cardiovascular risk factors, arterial hypertension, diabetes, hyperlipidemia, smoking and physical activity as well as cardiovascular comorbidities, apoplexia, peripheral atherosclerotic disease, aneurysm and arrhythmia. Rose questionnaire, CCS-, NYHA-classification and AHA heart failure stage are used to characterize the current cardiovascular status [9-12]. Family medical history of first degree relatives (parents, brothers, sisters and children) is obtained in detail for myocardial infarction, coronary revascularization (PCI/CABG), peripheral atherosclerotic disease, diabetes and obesity. The Paffenbarger questionnaire is used to obtain information about physical activity of the last 12 months [3]. In addition the food frequency questionnaire of the EPIC study [13] is answered by patients who are able to do so.

***Anthropometry, blood pressure and ankle-brachial-index***

Anthropometric measurements, determination of blood pressure and ankle brachial index (ABI) follow settings used in MONICA/KORA [13,14]. Anthropometry includes determination of weight, height, waist and hip circumference using SECA 701, 220 and 203 measuring systems (SECA, Germany). Weight is recorded with a precision of 0.1 kg, and height, waist and hip circumference to the nearest 0.5 cm. Blood pressure and heart rate are measured after a 10 minutes rest period with an automated oscillometric device OMRON 705IT (OMRON Healthcare, Germany) which meets the criteria for use in clinical trials and is recommended by the British Hypertension Society and Association for the Advancement of Medical Instruments [16]. Three measurements are performed in a sitting position at the right arm with 3-minutes delay, respectively. Blood pressure measurements for determination of ABI are performed after an at least 10-minutes rest period in a supine position. Systolic blood pressures of the right arm and both ankles are measured serial at twice by gold standard method Doppler ultrasound using sphygmomanometer cuffs and a handheld Doppler probe (Huntleigh Mini-Dopplex, Germany).

***Resting electrocardiogram and exercise stress test***

Resting electrocardiograms are recorded on the PC based 12-lead ECG system Del Mar Reynolds Medical CardioDirect 12 (Spacelabs Healthcare) in a supine position.

Exercise stress test is performed in study participants who are able to walk using a Cornell protocol for treadmill test [17]. Continuous blood pressure measurements and electrocardiograms are obtained during exercise and recovery. Standard criteria are used for termination of the stress test: significant chest pain, ventricular tachycardia, greater than 3 mm of ST-segment depression, decrease in systolic blood pressure of more than 20 mmHg, or until exhaustion. Experienced physicians read the stress test findings and results are classified as low, moderate, or high risk for CAD. Cardiopulmonary capacity is documented as the maximal obtained level of exercise in metabolic equivalents (METs).

***Echocardiography***

Echocardiography is performed according to AHA/ACC/ASE guidelines [18] following a standardized protocol [19]. Physicians and sonographers performing and interpreting cardiac ultrasound attended transthoracic echocardiography courses certified by the German Society for Ultrasound in Medicine (DEGUM) and German Society of Cardiology (DGK). They were trained for a standardized echocardiographic study protocol (Figure S1). Image recording is performed using GE Vivid 7 and Vivid q instruments with a 4.0-MHz transducer (GE Healthcare) while patient is in a left lateral decubitus position. Anatomical and functional characteristics are detected using real-time imaging B-mode, M-mode, continuous-wave (cw), pulsed-wave (pw) and tissue doppler imaging (TDI) in standard parasternal and apical views (Figure S1). Digital cine loops are acquired, respectively, and stored in raw data format to permit retrospective analyses. Standardized readings are performed at EchoPAC workstations (GE-Healthcare): chambers size, wall thickness, left ventricular mass, global left ventricular systolic function (ejection fraction), left ventricular diastolic function and valvular pathology (Table S1). Standardized image acquisition and storage in raw data format allow retrospective analyses of myocardial function using innovative image evaluation algorithms, e.g. speckle-tracking.

***Carotid ultrasound***

Ultrasound of carotid arteries is realized in all participants to study the wall morphology searching for signs of early atherosclerotic changes in the vessel wall and apparent subclinical atherosclerosis. Examinations are performed and interpreted by experienced and trained sonographers. High resolution B-mode ultrasound images are acquired using the GE Vivid 7 and Vivid q ultrasound platform with a 12.0-MHz linear-array transducer (GE Healthcare). Images are stored in raw data format for retrospective reading and measurement. A modified protocol from the Atherosclerosis Risk in Communities (ARIC) study [1] is used to acquire images of the common, bulb and internal segments of each carotid artery (Figure S2).

Examination and analysis follow the recommendations of the ‘Mannheim Carotid Intima-Media Thickness Consensus’ and the ‘American Society of Echocardiography’ with separate categorization of cIMT and plaque [20,21]:

(1) Carotid intima-media thickness (cIMT) is visualized as double-line on both walls of CCAs in a longitudinal view. It is formed by two parallel lines, which consist of the leading edges of two anatomical boundaries: the lumen-intima and media-adventitia interfaces. The mean and maximum of the combined thickness of the intimal and medial layer of the far wall of CCA are measured with a semiautomated border detection program (GE EchoPAC IMT). The detecting area of cIMT is defined as the distal 1 cm of the common carotid artery, immediately proximal to the origin of the bulb.

(2) Atherosclerotic carotid plaque is screened using transversal and longitudinal views in different axis of CCA, bulb and internal carotid artery (ICA). Plaque is defined as an echogenic thickening of intimal reflection that extends into the arterial lumen at least 0.5 mm or 50% of the surrounding cIMT value, or demonstrated an intimal + medial thickness of >1.5 mm. Plaque presence is documented as ‘present’ or ‘absent’ for each segment (CCA, bulb, ICA) of the right and left carotid artery, respectively.

***Coronary angiography***

Coronary wall irregularities and significant stenosis are detected by radiographic visualization of the coronary vessels after injection of radiopaque contrast media (Figure S3). Coronary angiography and left ventriculography are performed setting multiple projections following the standards of the institution. Movie clips of the angiogram are recorded digitally on CD-ROM according to DICOM standards and angiographic findings are interpreted immediately by a team of interventional cardiologists not directly involved in the study. The percentage narrowing of each coronary artery segment according to the AHA classification [22] is sized by visual assessment nearest to 0 %, 25 %, 50 %, 75 %, 90 %, 99 %, 100 % (total occlusion), as estimated from a comparison with the diameter of the normal reference level proximal to the lesion. Analyses are calculated for sets of patients with angiographic normal coronary arteries (*no CAD*), individuals with wall irregularities less than 50% luminal reduction (*CAD <50%)* and individuals with more than 50% luminal reduction (*CAD ≥50%)* in at least one coronary segment of the right, left anterior descending, or left circumflex system according to the AHA-classification (Figure S3). Gensini score index [23] is calculated and several further indices of the arteriographic extent of disease are selected and modified from the Coronary Artery Surgery Study (CASS) [2]: number of vessels diseased, number of segments diseased, number of proximal segments diseased, proximal arterial segment score. Significant LMCAD was defined as visual luminal reduction ≥ 50 % of the left main trunk. Plaques occured ostial, mid-shaft or at bifurcation into LAD and LCX (Figure S4).

***Sample size calculation***

We performed sample-size calculations to estimate the suitability of our data base for future planned genetic studies. We estimated required sample sizes for case-control comparisons and analyses of quantitative parameters (gene expression, proteomics). Assumed genetic effects varied between OR 1.2, 1.3, 1.5 or between differences in terms of standard deviation (10%, 20%, 30%), respectively (Table S2). Further assumptions are a power of 80%, a recessive major allele model of heritance, equal group sizes for case and controls and validity of Hardy-Weinberg Equilibrium (HWE). The finally available subjects (app. 2500 cases of stable obstructive CAD, 4000 cases of myocardial infarction, app. 2200 angiographically controls) are adequate for genetic analysis to detect low to intermediate effects (OR > 1.2 for categorical items and mean differences > 0.2*SD for linear parameters) for common SNPs (MAF > 20%). ***Genetic association analysis (chromosome 9p21)***

We genotyped SNPs rs10757274, rs2383206, rs2383207 and rs10757278 of the CAD-risk haplotypic block on chromosome 9p21 (Figure S5). Genotype quality was determined by exact test of Hardy-Weinberg Equilibrium (HWE). Call rate exceeded 97% for all SNPs. Haplotypes of SNPs rs10757274, rs2383206, rs2383207, rs10757278 were inferred using fastphase 1.2 [24]. Coronary atherosclerotic burden was adjusted to major risk factors of atherosclerosis (age, gender, smoking, diabetes, HDL- and LDL-levels adjusted for statin treatment) using logistic regression techniques. For association analysis, we assumed an additive model of heritability.

***References Supporting Information***

1. The Atherosclerosis Risk in Communities (ARIC) Study: design and objectives. The ARIC investigators. Am J Epidemiol 1989;129(4):687-702.

2. Ringqvist I, Fisher LD, Mock M, Davis KB, Wedel H, Chaitman BR, Passamani E, Russel RO, Alderman EL, Kouchoukas NT, Kaiser GC, Ryan TJ, Killip T, Fray D. Prognostic value of angiographic indices of coronary artery disease from the Coronary Artery Surgery Study (CASS). J Clin Invest 1983;71(6):1854-66.

3. Paffenbarger RS, Jr., Blair SN, Lee IM, Hyde RT. Measurement of physical activity to assess health effects in free-living populations. Med Sci Sports Exerc 1993;25(1):60-70.

4. Wichmann HE, Gieger C, Illig T; MONICA/KORA Study Group. KORA-gen--resource for population genetics, controls and a broad spectrum of disease phenotypes. Gesundheitswesen 2005;67 Suppl 1:S26-30.

5. Greiser KH, Kluttig A, Schumann B, Kors JA, Swenne CA, Kuss O, Werdan K, Haerting J. Cardiovascular disease, risk factors and heart rate variability in the elderly general population: design and objectives of the CARdiovascular disease, Living and Ageing in Halle (CARLA) Study. BMC Cardiovasc Disord 2005;5:33.

6. Riboli E, Kaaks R. The EPIC Project: rationale and study design. European Prospective Investigation into Cancer and Nutrition. Int J Epidemiol 1997;26 Suppl 1:S6-14.

7. Holdt LM, Beutner F, Scholz M, Gielen S, Gäbel G, Bergert H, Schuler G, Thiery J, Teupser D. ANRIL expression is qassociated with atherosclerotic risk at chromosome 9p21. Arterioscler Thromb Vasc Biol 2010;30(3):620-7.

8. Holle R, Giesecke B, Nagl H. PC-gestützte Datenerhebung als Beitrag zur Qualitätsicherung in Gesundheitssurveys: Erfahrungen mit DIAMON im KORA Survey 2000. Zeitschrift für Gesundheitswissenschaften 2000(8):165-173.

9. Hunt SA. ACC/AHA 2005 guideline update for the diagnosis and management of chronic heart failure in the adult: a report of the American College of Cardiology/American Heart Association Task Force on Practice Guidelines (Writing Committee to Update the 2001 Guidelines for the Evaluation and Management of Heart Failure). J Am Coll Cardiol 2005;46(6):e1-82.

10. Rose G, McCartney P, Reid DD. Self-administration of a questionnaire on chest pain and intermittent claudication. Br J Prev Soc Med 1977;31(1):42-8.

11. Sangareddi V, Chockalingam A, Gnanavelu G, [Subramaniam T](http://www.ncbi.nlm.nih.gov/pubmed?term=%22Subramaniam%20T%22%5BAuthor%5D), [Jagannathan V](http://www.ncbi.nlm.nih.gov/pubmed?term=%22Jagannathan%20V%22%5BAuthor%5D), [Elangovan S](http://www.ncbi.nlm.nih.gov/pubmed?term=%22Elangovan%20S%22%5BAuthor%5D). Canadian Cardiovascular Society classification of effort angina: an angiographic correlation. Coron Artery Dis 2004;15(2):111-4.

12. Bodegard J, Erikssen G, Bjornholt JV, Thelle D, Erikssen J. Possible angina detected by the WHO angina questionnaire in apparently healthy men with a normal exercise ECG: coronary heart disease or not? A 26 year follow up study. Heart 2004;90(6):627-32.

13. Kroke A, Klipstein-Grobusch K, Voss S, Möseneder J, Thielecke F, Noack R, Boeing H. [Validation of a self-administered food-frequency questionnaire administered in the European Prospective Investigation into Cancer and Nutrition (EPIC) Study: comparison of energy, protein, and macronutrient intakes estimated with the doubly labeled water, urinary nitrogen, and repeated 24-h dietary recall methods.](http://www.ncbi.nlm.nih.gov/pubmed/10500011) Am J Clin Nutr. 1999 Oct;70(4):439-47.

14. Keil U, Liese AD, Hense HW, Filipiak B, Döring A, Stieber J, Löwel H. Classical risk factors and their impact on incudent non-fatal and fatal myocardial infarction and all-cause mortality in southern Germany. Results from the MONICA Augsburg cohort study 1984-1992. Monitoring Trends and Determinants in Cardiovascular Diseases. Eur Heart J 1998;19(8):1197-207.

15. Lamina C, Meisinger C, Heid IM, Heid I, Löwel H, Rantner B, Koenig W, Kronenberg C for the KORA Study Group. Association of ankle-brachial index and plaques in the carotid and femoral arteries with cardiovascular events and total mortality in a population-based study with 13 years of follow-up. Eur Heart J 2006;27(21):2580-7.

16. Coleman A, Freeman P, Steel S, Shennan A. Validation of the Omron 705IT (HEM-759-E) oscillometric blood pressure monitoring device according to the British Hypertension Society protocol. Blood Press Monit 2006;11(1):27-32.

17. Rahimi K, Thomas A, Adam M, [Hayerizadeh BF](http://www.ncbi.nlm.nih.gov/pubmed?term=%22Hayerizadeh%20BF%22%5BAuthor%5D), [Schuler G](http://www.ncbi.nlm.nih.gov/pubmed?term=%22Schuler%20G%22%5BAuthor%5D), [Secknus MA](http://www.ncbi.nlm.nih.gov/pubmed?term=%22Secknus%20MA%22%5BAuthor%5D). Implications of exercise test modality on modern prognostic markers in patients with known or suspected coronary artery disease: Treadmill versus bicycle. Eur J Card Prev Rehabil 2006;13(1):45-50.

18. Cheitlin MD, Armstrong WF, Aurigemma GP, Beller GA, Bierman FZ, Davis JL, Douglas PS, Faxon DP, Gillam LD, Kimball TR, Kussmaul WG, Pearlman AS, Philbrick JT, Rakowski H, Thys DM, Antman EM, Smith SC Jr, Alpert JS, Gregoratos G, Anderson JL, Hiratzka LF, Faxon DP, Hunt SA, Fuster V, Jacobs AK, Gibbons RJ, Russell RO; ACC; AHA; ASE. ACC/AHA/ASE 2003 Guideline Update for the Clinical Application of Echocardiography: summary article. A report of the American College of Cardiology/American Heart Association Task Force on Practice Guidelines (ACC/AHA/ASE Committee to Update the 1997 Guidelines for the Clinical Application of Echocardiography). J Am Soc Echocardiogr 2003;16(10):1091-110.

19. Hagendorff A. Transthoracic echocardiography in adult patients - a proposal for documenting a standardized investigation. Ultraschall Med 2008;29(4):344-365; quiz 366-373.

20. Stein JH, Korcarz CE, Hurst RT, Hurst RT, Lonn E, Kendall CB, Mohler ER, Najjar SS, Rembold CM, Post WS. Use of carotid ultrasound to identify subclinical vascular disease and evaluate cardiovascular disease risk: a consensus statement from the American Society of Echocardiography Carotid Intima-Media Thickness Task Force. Endorsed by the Society for Vascular Medicine. J Am Soc Echocardiogr 2008;21(2):93-111; quiz 189-90.

21. Touboul PJ, Hennerici MG, Meairs S, Adams H, Amarenco P, Bornstein N, Csiba L, Desvarieux M, Ebrahim S, Fatar M, Hernandez R, Jaff M, Kownator S, Prati P, Rundek T, Sitzer M, Schminke U, Tardif JC, Taylor A, Vicaut E, Woo KS, Zannad F, Zureik M. Mannheim carotid intima-media thickness consensus (2004-2006). An update on behalf of the Advisory Board of the 3rd and 4th Watching the Risk Symposium, 13th and 15th European Stroke Conferences, Mannheim, Germany, 2004, and Brussels, Belgium, 2006. Cerebrovasc Dis 2007;23(1):75-80.

22. Austen WG, Edwards JE, Frye RL, [Gensini GG](http://www.ncbi.nlm.nih.gov/pubmed?term=%22Gensini%20GG%22%5BAuthor%5D), [Gott VL](http://www.ncbi.nlm.nih.gov/pubmed?term=%22Gott%20VL%22%5BAuthor%5D), [Griffith LS](http://www.ncbi.nlm.nih.gov/pubmed?term=%22Griffith%20LS%22%5BAuthor%5D), [McGoon DC](http://www.ncbi.nlm.nih.gov/pubmed?term=%22McGoon%20DC%22%5BAuthor%5D), [Murphy ML](http://www.ncbi.nlm.nih.gov/pubmed?term=%22Murphy%20ML%22%5BAuthor%5D), [Roe BB](http://www.ncbi.nlm.nih.gov/pubmed?term=%22Roe%20BB%22%5BAuthor%5D). A reporting system on patients evaluated for coronary artery disease. Report of the Ad Hoc Committee for Grading of Coronary Artery Disease, Council on Cardiovascular Surgery, American Heart Association. Circulation 1975;51(4 Suppl):5-40.

23. Gensini GG. A more meaningful scoring system for determining the severity of coronary heart disease. Am J Cardiol 1983;51(3):606.

24. Scheet P, Stephens M. A fast and flexible statistical model for large-sclae population genotype data: applications to inferring missing genotypes and haplotypic phase. Am J Hum Genet 2006;78:629-644.
